# Supplementary material for: The Bph45 Gene Confers Resistance against Brown Planthopper in Rice by Reducing the Production of Limonene
Source: Int J Mol Sci. 2023 Jan 16;24(2):1798. doi: 10.3390/ijms24021798 (PMC9863282; doi:10.3390/ijms24021798)
Supplement: Supplementary file 1 [file ijms-24-01798-s001.zip › ijms-2130500-supplementary.pdf]

Table S1. Differential VOCs between TNG71 and TNG71-*Bph45* revealed by GC-MS.

Name of compound[a]	Retention time (min)	Relative amount in each TNG71 experiment (%)					Count in TNG71	**Avg. amount (%) in TNG71**	Relative amount in each TNG71-Bph45 experiment (%)					Count in TNG71-Bph45	**Avg. amount (%) in TNG71-Bph45**	p value (Student's unpaired t-test)	Summary (x indicates no differences between two lines); blue font indicates a difference
		#1[b]	#2[b]	#3[b]	#4[b]	#5[b]			#1[b]	#2[b]	#3[b]	#4[b]	#5[b]				
Carbamic acid, monoammonium salt	0.86	0.00	3.31	0.00	0.00	0.00	1	**3.31**	0.00	4.05	6.33	5.62	5.59	4	**5.40**	0.0243	significantly more in TNG71-Bph45
Acetone	1.57	1.65	2.02	5.66	2.21	2.92	5	**2.89**	1.10	3.17	6.40	2.45	5.61	5	**3.75**	0.5036	x
Acetic acid	2.31	1.01	0.00	2.86	1.35	1.41	4	**1.66**	0.00	0.00	0.00	1.02	1.68	2	**1.35**	0.2105	x
Cyclopentane, methyl-	2.95	0.46	0.00	0.00	0.00	0.00	1	**0.46**	0.00	6.70	15.42	0.00	1.48	3	**7.87**	0.1548	x
Isopropoxycarbamic acid, ethyl ester	4.16	0.00	1.05	0.00	0.00	0.00	1	**1.05**	1.38	2.34	0.00	0.00	0.00	2	**1.86**	0.3368	x
Furan, 2-ethyl-	4.61	0.00	1.02	1.13	0.00	0.00	2	**1.08**	0.00	0.00	0.00	0.00	0.90	1	**0.90**	0.4572	x
3-Pentanone	4.65	1.59	1.78	1.39	0.00	0.00	3	**1.59**	0.90	2.07	3.92	0.00	1.42	4	**1.66**	0.3836	x
2,5-Dimethylfuran	5.01	1.44	0.90	1.00	0.58	0.00	4	**0.98**	1.11	0.62	0.00	0.99	0.52	4	**0.81**	0.6732	x
Toluene	7.42	6.94	5.14	5.90	3.96	4.00	5	**5.19**	4.61	3.87	6.40	3.08	3.21	5	**4.23**	0.2851	x
3-Methylthiophene	7.97	2.73	1.51	3.04	2.23	2.13	5	**2.33**	0.00	0.00	0.00	0.00	0.00	0	**0.00**	0.0000	specific to TNG71
Methyl 2-methylbutyrate	8.09	0.00	0.00	0.00	0.00	0.00	0	**0.00**	3.35	2.82	3.63	3.44	2.94	5	**3.24**	0.0000	specific to TNG71-Bph45
4-Pentenal, 2-methyl-	8.85	1.42	1.97	0.00	0.00	0.00	2	**1.70**	0.82	0.00	0.00	0.00	0.00	1	**0.82**	0.2902	x
3-Hexenal	9.05	0.00	0.00	0.95	0.00	0.00	1	**0.95**	0.00	0.97	0.00	0.00	1.44	2	**1.20**	0.4388	x
2-Hexenal or 3-Hexen-1-ol, (E)-	11.55	6.05	8.15	4.99	4.07	1.74	5	**5.00**	1.96	0.63	1.43	3.10	3.45	5	**2.11**	0.0404	significantly more in TNG71
Ethylbenzene	11.80	1.77	2.01	1.47	2.33	1.80	5	**1.88**	0.93	0.89	1.22	1.83	1.12	5	**1.20**	0.0154	significantly more in TNG71
p-Xylene	12.18	6.30	0.00	0.00	9.94	9.13	3	**8.46**	4.35	3.86	6.23	8.71	3.67	5	**5.37**	0.9042	x
2-Heptanone[c]	13.16	3.66	5.23	4.07	2.42	2.98	5	**3.67**	12.91	11.99	9.11	2.00	4.90	5	**8.18**	0.0679	x
o-Xylene	13.28	4.49	0.00	0.00	4.73	3.98	3	**4.40**	0.00	0.00	0.00	4.76	0.00	1	**4.76**	0.2760	x
2-Heptanol[c]	13.60	2.66	3.11	1.84	0.00	0.00	3	**2.54**	3.38	3.53	2.48	0.00	2.12	4	**2.88**	0.4165	x
(1R)-2,6,6-Trimethylbicyclo[3.1.1]hept-2-ene	15.14	0.00	0.49	1.19	0.00	0.91	3	**0.86**	0.89	0.89	2.19	1.71	2.45	5	**1.63**	0.0247	x
Octane, 2,2,6-trimethyl-	16.29	2.38	0.00	0.00	2.78	3.35	3	**2.83**	1.65	0.00	0.00	2.84	0.00	2	**2.25**	0.4082	x
5-Hepten-2-one, 6-methyl-	17.53	1.03	0.71	0.77	1.71	2.15	5	**1.27**	0.77	1.04	1.13	1.52	2.06	5	**1.30**	0.9359	x
.beta.-Myrcene	17.69	1.81	2.34	1.14	1.27	1.49	5	**1.61**	3.31	1.21	0.00	1.54	1.02	4	**1.77**	0.7475	x
Mesitylene	17.84	1.97	0.00	1.57	0.00	0.00	2	**1.77**	0.00	1.05	2.20	0.00	1.63	3	**1.63**	0.6759	x
Tridecane	18.01	0.00	0.00	0.00	0.00	1.09	1	**0.55**	0.55	0.00	0.00	0.76	0.00	2	**0.66**	0.8744	x
Octanal	18.20	0.96	0.00	0.00	0.00	0.88	2	**0.92**	0.00	0.58	0.00	0.81	0.00	2	**0.69**	0.7567	x
Decane, 2,2-dimethyl-	19.04	0.00	0.71	0.00	1.07	0.00	2	**0.89**	0.64	0.00	0.00	1.08	0.51	3	**0.74**	0.7754	x
Benzene	19.18	2.34	3.38	1.48	4.14	3.75	5	**3.02**	3.23	0.73	0.00	2.84	1.00	4	**1.95**	0.1039	x
Limonene	19.36	6.16	7.84	6.76	9.80	8.49	5	**7.81**	4.97	2.62	1.82	5.32	3.41	5	**3.63**	0.0020	significantly more in TNG71
3-Methyl-5-propylnonane	19.52	0.77	1.27	2.23	0.00	1.22	4	**1.37**	0.00	0.00	0.00	0.00	0.00	0	**0.00**	0.0164	specific to TNG71
Eucalyptol	19.52	0.00	0.00	0.00	0.00	0.00	0	**0.00**	2.45	2.34	2.54	0.00	4.35	4	**2.92**	0.0097	specific to TNG71-Bph45
2,5-Hexanedione, 3,4-dimethyl-	19.86	1.43	1.51	1.54	1.75	1.89	5	**1.62**	0.00	1.34	1.08	1.51	1.66	4	**1.40**	0.1389	x
1-Octen-3-ol	20.51	0.00	2.42	0.00	0.00	0.00	1	**2.42**	1.64	2.37	0.00	0.00	3.98	3	**2.66**	0.2491	x
Undecane, 6-methyl-	21.82	0.00	0.00	0.00	0.66	1.00	2	**0.83**	0.00	0.00	0.00	0.88	0.00	1	**0.88**	0.5825	x
2-Nonanone	21.93	2.85	3.30	2.96	1.51	2.39	5	**2.60**	4.40	3.25	1.79	1.99	2.73	5	**2.83**	0.6934	x
Linalool[d]	22.24	4.09	5.52	18.4	1.45	2.24	5	**6.34**	6.62	10.69	5.66	2.58	4.84	5	**6.08**	0.9399	x
Nonanal	22.43	1.56	1.02	1.55	1.59	1.66	5	**1.47**	0.77	1.13	0.75	1.60	1.56	5	**1.16**	0.1860	x
2,5-Dihydroxybenzaldehyde	22.66	0.75	1.57	1.06	0.69	1.20	5	**1.05**	0.55	0.53	0.00	0.74	0.00	3	**0.61**	0.0146	x
(E)-4,8-Dimethylnona-1,3,7-triene	22.90	8.08	8.71	9.20	7.54	9.98	5	**8.70**	5.96	5.74	0.00	7.21	5.97	4	**6.22**	0.0240	significantly more in TNG71
13-Methyltetradecanal	24.99	0.46	0.00	0.00	1.10	0.00	2	**0.78**	0.00	0.00	0.00	1.02	0.00	1	**1.02**	0.7239	x
Benzoic acid, ethyl ester	25.15	2.83	2.97	1.88	0.00	0.00	3	**2.56**	3.22	1.72	2.84	0.00	0.00	3	**2.59**	0.9821	x
Methyl salicylate	26.12	6.74	6.18	5.45	1.93	1.80	5	**4.42**	5.73	6.97	3.53	3.25	3.82	5	**4.66**	0.8558	x
Decanal	26.35	3.46	1.77	1.82	4.28	4.09	5	**3.08**	1.65	2.20	1.69	4.54	3.40	5	**2.69**	0.6306	x
Cyclohexasiloxane, dodecamethyl-	30.49	0.00	1.49	1.00	3.63	2.75	4	**2.22**	0.55	0.00	0.00	2.93	2.60	3	**2.03**	0.5549	x
Copaene	32.64	0.79	0.86	1.31	1.83	2.18	5	**1.39**	0.81	0.53	1.08	0.95	1.21	5	**0.92**	0.1445	x
Tetradecane	33.04	1.22	2.05	1.77	2.04	2.48	5	**1.91**	1.20	1.45	0.00	0.00	2.58	3	**1.74**	0.1395	x
Caryophyllene[d]	34.18	0.57	1.14	1.43	3.85	2.81	5	**1.96**	0.83	0.57	1.48	2.63	2.28	5	**1.56**	0.5915	x
cis-.alpha.-Bergamotene	34.51	0.00	0.00	0.00	0.98	0.00	1	**0.98**	0.58	0.00	0.89	0.97	0.00	3	**0.81**	0.3376	x
5,9-Undecadien-2-one	34.90	0.00	0.00	0.00	0.00	1.17	1	**1.17**	0.00	0.55	0.00	0.00	0.49	2	**0.52**	0.9238	x
1,4,7,-Cycloundecatriene	35.30	0.00	0.00	0.00	0.87	1.17	2	**1.02**	0.00	0.00	0.00	0.80	0.00	1	**0.80**	0.4345	x
Benzene	35.89	1.12	1.07	0.00	1.73	1.91	4	**1.46**	1.75	0.70	1.31	2.78	0.98	5	**1.50**	0.5172	x
2,4-Di-tert-butylphenol	36.55	0.59	0.00	0.00	0.00	0.51	2	**0.55**	0.00	0.00	2.29	0.00	0.87	2	**1.58**	0.4057	x
gamma.-Muurolene	36.86	0.00	0.00	0.00	0.00	0.92	1	**0.92**	1.29	0.00	0.94	1.86	1.08	4	**1.29**	0.0429	significantly more in TNG71-Bph45
Naphthalene	37.05	0.00	0.00	0.00	1.64	0.00	1	**0.55**	3.23	1.08	2.21	4.36	2.72	5	**2.72**	0.0055	significantly more in TNG71-Bph45
Nonadecane	38.29	0.60	0.56	0.00	3.39	0.00	3	**1.52**	0.00	0.00	0.00	0.00	0.00	0	**0.00**	0.1888	specific to TNG71
Cyclooctasiloxane, hexadecamethyl-	39.38	0.58	1.29	0.00	0.00	0.00	2	**0.93**	0.00	0.00	0.00	1.08	0.00	1	**1.08**	0.6509	x
Heptadecane, 2-methyl-	39.96	2.70	2.64	1.17	0.00	3.41	4	**2.48**	0.00	1.22	0.00	0.00	0.00	1	**1.22**	0.0302	significantly more in TNG71
Eicosane	40.17	0.00	0.00	0.00	1.95	0.00	1	**1.95**	3.50	0.00	0.00	0.00	0.76	2	**2.13**	0.5708	x
2-Pentadecanone, 6,10,14-trimethyl-	42.00	0.00	0.00	0.00	1.02	1.00	2	**1.01**	0.45	0.00	0.00	0.89	0.00	2	**0.67**	0.6696	x
Total %		100	100	100	100	100			100	100	100	100	100				

[a] Compounds that appeared only once in ten GC-MS analyses or twice but just once in each side were removed. Compounds present at amounts less than 1% in both original samples were also removed.

[b] Experiment number.

[c] Reported as repellent to BPH by Lu J. et al. (2014) Mol. Plant. 7, 1670–1682.

[d] Reported as an attractant or repellent to BPH by Xiao, Y. et al. (2012) Ecology Letters, 15(10): 1130-1139.

Table S2. Rice limonene synthase genes and PCR primers.

Gene name	Locus name	Forward primer	Reverse primer	Product (bp)	Reference
Osubq5	Os01g0328400	ACCACTTCGACCGCCACTACT	ACGCCTAAGCCTGCTGGTT	69	Jain et al. (2006) BBRC 345: 646–651
OsTPS19	Os04g0340300	CACGAGGTTGCTCTCCACTT	GACAAGGAGGTGAGCTGCAT	161	Chen et al. (2018) Plant Biotechnology Journal 16, 1778–1787.
OsTPS20	Os04g0341500	same as OsTPS19	same as OsTPS19	161	Lee et al. (2016) Protoplasma 253(3):683-690.

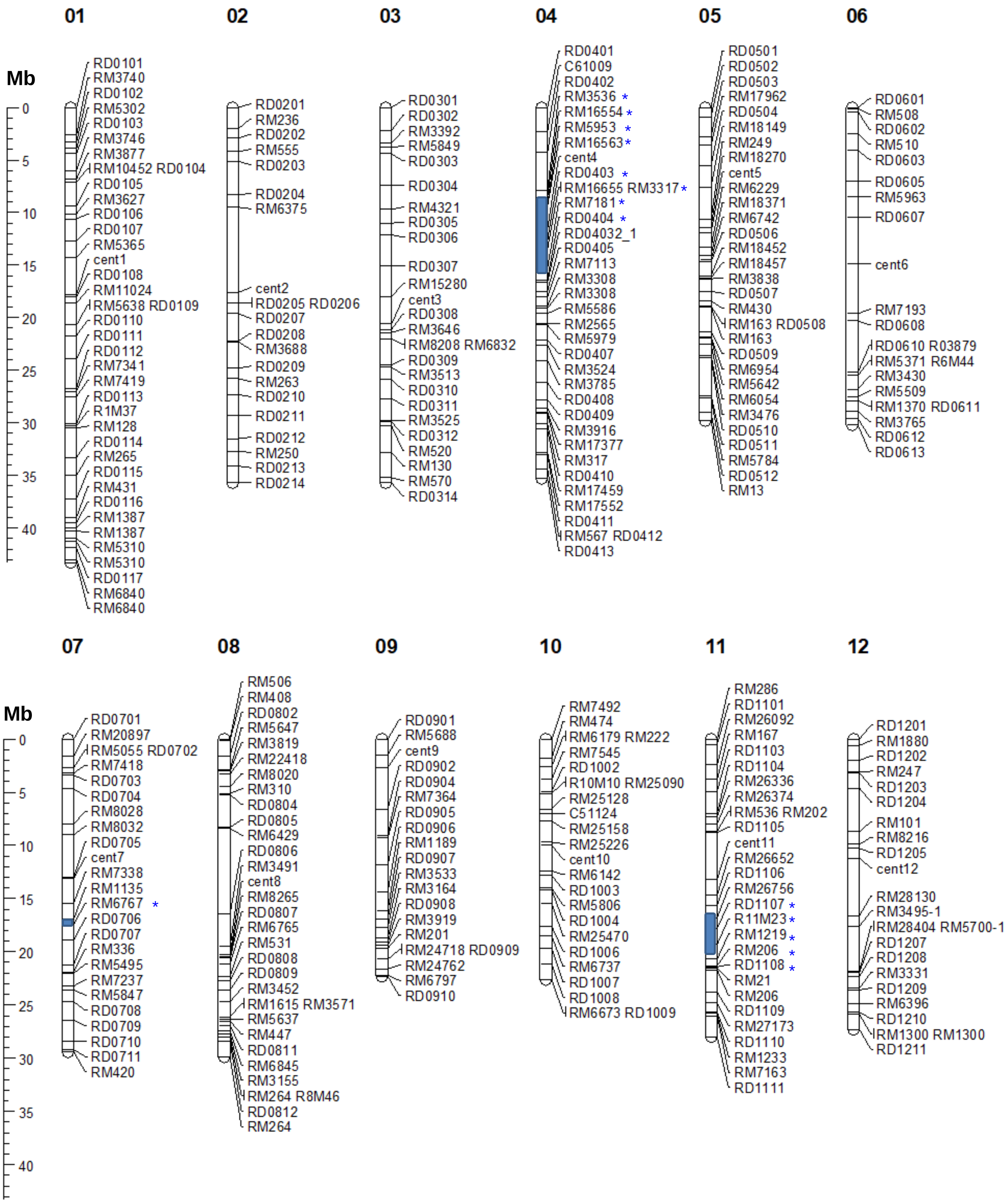

Figure S1. Chromosome-wise characterization of the recurrent genome recovery of TNG71-*Bph45*. 134 InDel (RD as prefixes) and 173 SSR (RM as prefixes) markers were identified by parental polymorphism survey between 'TNG71' and 'Oryza nivara (IRGC 102165)', and were used to evaluate the recurrent genome recovery of TNG71-*Bph45*. All markers were assigned to the linkage map of Nipponbare /Kasalath based on the primer sequences of markers by blasting against PACs/BACs, which were integrated into the linkage map by IRGSP. Cent* indicate centromere. Chromosomal regions and markers which polymorphic between TNG71-*Bph45* and TNG71 were indicated by blue boxes and blue stars, respectively.

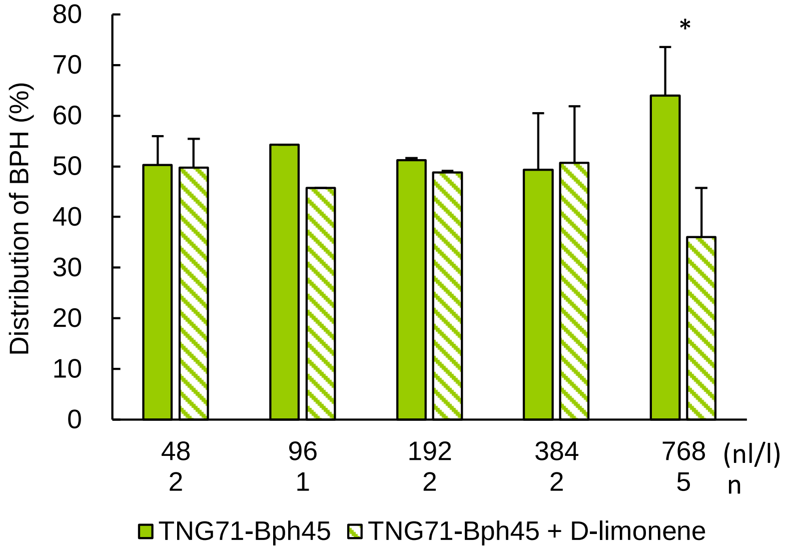

Figure S2. Repellence of BPH by limonene in Y-maze tests.
As in Figure 2, the relative distribution of BPH in a Y-maze test was determined using various amounts of D-limonene in the presence of TNG71-*Bph45* rice. Results of independent replicates (as indicated) were analyzed using Student's paired *t*-test. *$p<0.05$, **$p<0.01$.

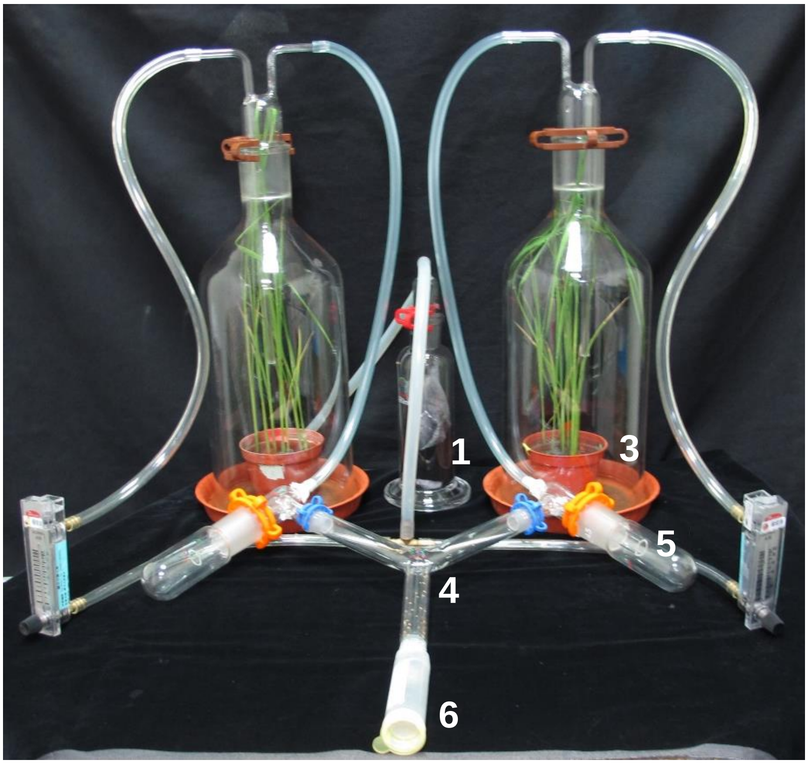

1: Charcoal filter (connected to the air pump)
2: Air control valve
3: Plant VOC
4: Y tube
5: BPH trap
6: BPH source

Figure S3. Y-maze olfactometer.
The air was supplied from #1 to #6 and was controlled by the valve (#2).
